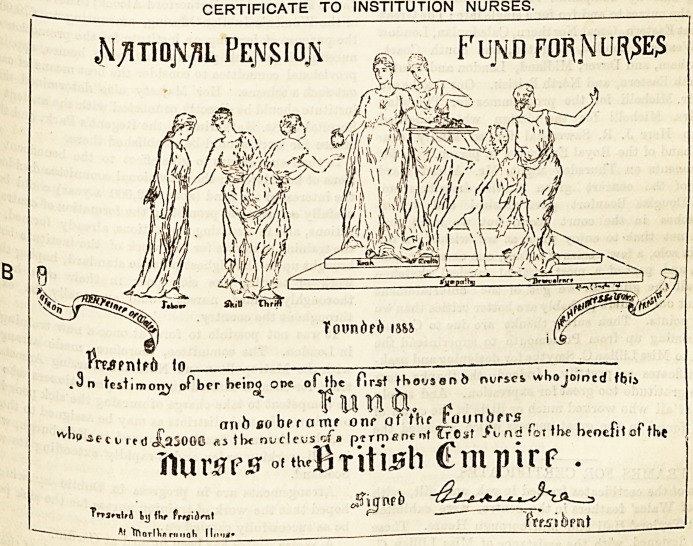# Extra Supplement—The Nursing Mirror

**Published:** 1890-07-12

**Authors:** 


Being the Extra Nursing Supplement of "The Hospital" Newspaper.

All Contributions for this Supplement should be addressed to the Editor, The Hospital, 140, Strand, London, W.C., and should have the word "Nursing" plainly written in left-hand top corner of the envelope.

### En Passant.

**Bucks Nursing Home.**—The twentieth annual report of this Home is out. No less than 44 cases were admitted during the year, 23 from Buckingham, and 21 from the villages round—of these 17 were cured, 16 relieved, 3 left unimproved, 3 died, 5 remained in the Home. The total number of days spent by patients in the Home during the year was 1,511. The ordinary expenses of the Home for the same period amounted to £310 15s. 2d., exclusive of rates and taxes. The average cost of each patient, including establishment charges, was £7 1s. 3d., average daily cost, 4s. 1½d. The payments made by patients, or by friends on their behalf, averaged 8⅝d. per patient per day. The Home, in common with all charitable institutions, has lost a true friend by the decease of the Right Hon. Lord Addington.

**Short Items.**—We hope the First Thousand saw last Saturday's *Daily Graphic* and this week's *Ladies' Pictorial*.—The Correctional Tribunal of L'Orient lately sentenced a midwife to six months' imprisonment. She was accused of having caused in one month, through neglect of the necessary precautions, the death of seven of her patients, who succumbed to puerperal fever.—Helen Cusa, a Moldavian Princess, widow of Prince Alexander Cusa, who left her over £100,000, has decided to devote the whole of her yearly income to charities. She is a nurse in a children's hospital at Jassy, to which she contributes £1,000 yearly.—The Methodists are starting an order of deaconesses, who are to be trained in district nursing.—Sister Rose Gertrude writes to acknowledge £121 from the readers of the *Pall Mall Gazette*.—Emma Jane Pike has patented an improved bronchitis kettle.—Messrs. Southall, whose hygienic inventions are familiar to all nurses, have just patented a combined stocking suspender, which should prove valuable.

**Pay or be Paid?**—This is a question for probationers which is seriously occupying the attention of American medical journals. Here is the opinion of the *New York Medical Journal*: "The benefits to the sick conferred by the training schools are incalculable. The community benefits greatly by the work of the nurses, and the better the nurses the greater the benefit. All this is true, but it is equally true in the case of physicians, architects, lawyers, plumbers, and almost anybody from whose work the community benefits. We doubt, however, if the proposition to pay persons to learn the plumbing trade, for example, would meet with much favour. We do not hear of any desire to pay medical students for studying their profession, which requires much the same qualities as that of nursing. The only excuse for paying the students of training schools would be the impossibility, without doing so, of finding persons fit for the profession. This means simply either that the demand greatly exceeds the supply, or that none but the very poor have the needed ability. Both these propositions are absurd; 1,306 applications were received last year at the Bellevue School, where there were only 31 vacancies. The supply is sufficient. It would not be hard to fill the school with good students if no pay was offered." J. West Roosevelt, M.D., also writes that the hospital manager who spends the hospital's money to educate someone is guilty of a breach of trust. He proposes that scholarships might be offered for poor nurses unable to pay for their teaching.

**Dundee Nursing Society.**—This society has lately secured a small villa as a nursing home, which has been formally opened by ex-Provost Moncur. Shortly after the society was formed the services of two nurses, viz., Miss M'Kay and Miss Byrte, were secured from the Queen Victoria Institute, Edinburgh, with which, we understand, the society is affiliated. The necessity for such a society then became apparent, for since March no fewer than 100 cases have been attended, 31 of the patients being regularly visited until they recovered. The nurses' home opened on June 26th has accommodation for six or eight nurses. It starts free of debt, and the friends of the society will thus be able to concentrate their efforts on the maintenance of an efficient staff of nurses. Miss Peter was present at the opening.

**Ladies as Nurses.**—Mrs. Lynn Linton is well known as a clever but prejudiced woman, especially where her own sex is concerned; her article on nursing, then (of which she has no experience), which lately appeared in the *St. James's Gazette* is scarcely worth quoting, were it not always well for us to know what curious motives some people can ascribe to our acts. "This hospital nursing, for example, which has become quite a fashionable craze among ladies, at first sight looks like the nineteenth-century expression of the old spirit of devotion which made the mediæval penitents and nuns. It may be so; but we must not forget that to the restless temper of the times, hospital nursing is more agreeable than a home life of peaceful monotony; and that to those who are not well endowed with the world's goods it offers an income for the present and by no means poor matrimonial chances for the future. To the outsider, too, the fact that those ladies with sufficient means who have devoted themselves to hospital nursing as a profession have taken the place of women who are in as much need of food as the poor patients themselves, is not very conducive to enthusiasm." *There you are, dear readers! That is the estimation in which you are held by a part, a very small part luckily, of your own sex.* But vilification is ever the meed of the reformer, and so long as our conscience does not accuse us we can withstand all attacks from without.

**The Garden Party at Marlborough House.**—The occasion of the reception at Marlborough House of the representatives of the first thousand nurses who joined the Pension Fund by the Prince and Princess of Wales was unique and unprecedented. It is the first time that women have successfully combined to establish an institution of great financial strength for their own benefit and that of their fellow-workers. It was the first occasion, we understand, on which the representatives of any philanthropic institution have been invited to Marlborough House, and, we believe, the first time in the history of this country, when the Heir-apparent and his Consort have specially identified themselves with the great army of workers who devote their lives to the care of the sick. It is the first time in the history of any benevolent institution in this country that £40,000 has been raised by subscription from a number of people without a large expenditure on printing, postages, advertising, and other similar expenses. It is certainly the first time in the history of the world that the memory of a citizen—the late Mr. Junius S. Morgan—has been perpetuated by the representatives of the class he strove to benefit in his lifetime, by the spontaneous subscriptions of nearly £2,500 collected in consequence of a paragraph in one newspaper (The Hospital), and without the usual expenditure, or, indeed, any publicity in the ordinary acceptation of that term. It is fitting, therefore, that everything connected with so memorable an event should have passed off to the complete satisfaction of everybody concerned.

### The Fête of the First Thousand.

### THURSDAY NIGHT.

In cloak and bonnet, or in apron and cap, came crowds of nurses to Merchant Taylors' Hall. The high hall was dim and mysterious, and the bright scarlet uniforms of four sergeants of the Royal Marines stood forth boldly amongst the quieter uniforms of the nurses. The drilling of the nurses went on to the music of gentle laughter and chatter; they fell into their places marvellously quickly to the evident delight of the sergeants, and answered to their numbers promptly and well. The *Daily Graphic* artist leant against the wall at one end of the room, and when a sergeant marched a company of nurses right up to him, and halted them within an inch of his toes, he dropped his eyeglasses and looked up, —and was not a whit abashed! He promptly asked permission to sketch all in the front row, and they as promptly acceded, and so with amusing incidents to cheer this somewhat trying period, the business of the evening was got through. About eight o'clock the guests began to arrive, the band struck up in the central hall, and concerts and entertainments began in the different reception rooms. It was easy to tell you were in the city, and in the home of one of its rich companies, for whichever way you walked you were sure to find yourself in a refreshment room! And the champagne cup was good and the strawberries were sweet, and those nurses who were somewhat tired by the drilling were soon refreshed and rested. At nine o'clock a crowd gathered in the great hall to hear Mr. Jonathan Hutchinson's address. Mr. Burdett took the chair, and made a few preliminary remarks. He was able to state that, thanks to the generosity of the different railway companies, every nurse present who had come more than twelve miles by rail, could have the difference between a single and a return ticket returned to her by showing her invitation card to Marlborough House. This pleasant announcement was received with applause. Furthermore, Mr. Burdett went on to say that the sum raised for a Benevolent Fund by those present in the course of a short six weeks, had far exceeded the anticipations of the Pension Fund authorities; the thanks of all were due to the nurses who had worked so well in this cause. Mr. Burdett welcomed all present cordially, told them it was their evening, and they were to do their best to enjoy themselves. Then followed an earnest and thoughtful speech by Mr. Jonathan Hutchinson, President of the Royal College of Surgeons, who paid the nurses the just compliment of addressing them as a cultivated audience. He began by an apology for having no speech prepared, and said Mr. Thomas Bryant, who was on the platform, was far better fitted than himself to speak to those assembled. "But as a surgeon," he continued, "I can but say that you have our hearty sympathy in your work, and we wish you all success; we acknowledge the importance of your duties, and the ever-increasing demand upon your skill. We live in an age of great activity and considerable knowledge; everything is moving forward towards vast improvement. I regard the success of this Pension Fund as an important sign of the times, for it shows what women can do, and will do, in these days. We have now a lady Senior Wrangler, and the education of all women is advancing, they are also encouraged to undertake many spheres of labour from which they were formerly debarred. Even that class of society which was in the past forbidden to work are now censured if they remain drones. It is an age of pay and an age of patience. Two of the foremost writers of the century have been women—George Eliot and Mrs. Barrett Browning. The works of the latter might be taken as the gospel of the women of to-day; apt quotations from her poems might be gathered in hundreds, but there are two I should specially like to bring before you to-night: first—

'Free men freely work;
Who fears God, fears to sit at ease.'

Secondly:—

'It takes a soul to move a body.'

These are very beautiful thoughts, especially for nurses. But so far as we know, without going deeply into psychological questions, it also takes a body to move a soul, and, therefore, it is that we must take care of our bodies. If our physical health fails, our mind fails, our abilities grow weak. And 'we have this treasure in earthen vessels,' so that for the sake of all that is best in us, of all that raises us to our highest achievements, we must guard the earthen vessel and its rich treasure within. There is nothing which so weakens both mind and body as anxiety; there is nothing which so hinders our powers of work. And what this Pension Fund has done for you is to remove all fear for your future; you can rest secure in the knowledge that the latter part of your life is provided for; you need never be oppressed in mind and body by this terrible gnawing anxiety. Men have long been accustomed to these insurance societies, and they have been the comfort of our lives. I recommend very strongly to you this system of insurance, especially as your fund is managed by those whose business capacities are of the highest order, and you can trust them with perfect confidence.' These were the main points in Mr. Hutchinson's speech, much of which was unfortunately lost to those on the outside of the crowd. When it was over the nurses dispersed once more to the different entertainments, finally re-assembling about eleven o'clock to propose a vote of thanks to Mr. Burdett, and to give a hearty round of applause to Mr. Clifford. Amongst those present were Mr. Rawlings, Mr. Thomas Bryant, Dr. Billings of Baltimore, Dr. Steele, Dr. George Potter, Mr. Rathbone, M.P., Miss Hutchinson, Mrs. Dacre Craven, resplendent in her many orders won on foreign nursing fields, and the matrons of numerous hospitals, including those of the London, Paddington Infirmary, Winchester, Gloucester, &c.

---

### FRIDAY MORNING.

It is not easy to sit down and write a description of the reception at Marlborough House after reading the very picturesque reports which appeared in the daily papers—particularly in the *Daily Graphic*. But perhaps eyes accustomed to nursing details saw a few special incidents which passed unnoticed by others. The gates of Marlborough House were perfectly besieged by an army of nurses about noon, and the crowd watched them entering with great interest. Most of the nurses were in indoor uniform, but a few wore cloaks and veils. Punctually at one o'clock the Princess of Wales, wearing the Royal Red Cross and the Order of St. John, appeared on the steps of the conservatory adjoining Marlborough House, and with her were the Prince of Wales, Princesses Victoria and Maud, the Duke of Clarence and Avondale, the Duchess of Beaufort, Lady Rosebery, Lady Rothschild, and a few others. The nurses were grouped on the great lawn opposite the windows and marched forward in excellent order to receive their certificates. The *Daily News* called it "the prettiest sight of the season," and truly, thanks to the fine weather, and the drilling, and excellent arrangements throughout, all went exceedingly well. Purses were deposited on a table beside the Princess, and it was pleasant to see with what care some of the little satin bags had been embroidered with initials. All the Mildmay nurses in their blue gowns and brown bonnets and veils came up together; but as a rule the uniforms were mixed, and the neat bibs of the Lincoln nurses, the frilled muslin caps of the St. John the Divine, and the Galatea frocks of Winchester were dispersed amongst private uniforms—not so well known, but equally becoming.

There were, too, Army Sisters in their distinctive red capes, but the Navy was not represented, except by the Seamen's Hospital.

The Prince of Wales' Address.

When all the certificates had been distributed the Prince of Wales bade the nurses draw nearer, and then addressed them in the following terms :—

Nurses, it has afforded the Princess and myself much pleasure to receive you here to-day. I understand that you come from nearly one hundred and fifty hospitals, infirmaries, asylums, nursing and other institutions in the United Kingdom, and that many of you are working on your own account as private nurses. One nurse present has come all the way from Gibraltar, and letters and purses have been received from members of the first thousand, who are working as nurses in Australia, Canada, Constantinople, Malta, Egypt, the United States, France, and Italy. One nurse at the Citadel Hospital in Cairo has sent an Egyptian purse containing £10, and another nurse has forwarded a purse from Natal. All this shows that the Pension Fund for Nurses is rightly named National. What, then, is the National Pension Fund for Nurses, and who are the first thousand nurses? Three years ago four of the leading merchants of the City of London deposited £20,000 with the Court of Chancery to enable the National Pension Fund to be incorporated under the Insurance Companies Acts, with the necessary financial guarantees. These gentlemen set the whole nursing body a lesson in thrift, and attached to the deposit a condition that unless 1,000 nurses invested their savings in the Pension Fund before January 1st, 1890, the £20,000 was to be returned and the Fund wound up. In less than twelve months from the date that the Council began to issue policies—August, 1888—1,000 were applied for, and the conditions were thus fulfilled. The four benefactors there and then made a gift of the £20,000, and of the fund specially allocated £5,000 was to be invested at compound interest for the sole benefit of the first 1,000 nurses who had, by their thrift and foresight, secured so splendid a sum for the benefit of the whole nursing body for ever. The National Pension Fund has greatly prospered since. Nurses continue to join it at an average of ten a week. Other friends have sent gifts to the Pension Bonus Fund, which amounts to nearly £40,000, and the invested funds to £67,000, the difference representing the investment by the nurses of their savings. But the National Pension Fund has done more than this. Heretofore, if a nurse fell ill or became disabled through no fault of her own, her position was often a very painful one, and many nurses under such circumstances formerly had no refuge but the workhouse. The philanthropists who have founded the Pension Fund have done much to prevent any such catastrophe befalling any nurse in future. All who join it will at once ensure safety for themselves and respect for the noble calling to which they belong. And already several who have not joined have been saved from distress through its agency. An old nurse, aged seventy, whose savings were all lost through a bad investment, is now receiving £30 per annum, and a nurse of thirty-five, who trod upon a needle while at work in a hospital ward, and had to have her foot amputated, is now upon the fund. But a third case is almost the saddest of all. A young nurse, aged twenty-one, joined the fund for sick pay and pension about two years ago. She was in good health at the time, and might have argued, as so many young nurses do argue, that she was not likely to require any help for many years to come, and that she might spend all her earnings, as it was too early to begin to save. Fortunately for her, she was much too thrifty to do anything of the kind, and when she became paralysed a few months after she joined, she received sufficient sick pay to provide her with necessary comforts and nursing during the few months of life that remained to her. There was no one who took a kindlier, a more generous interest in such cases as I have mentioned than the late Mr. Junius S. Morgan, who was one of the four original donors, and who gave altogether £10,000 to the National Pension Fund. It is, therefore, an additional pleasure and satisfaction both to the Princess and myself that the first thousand nurses who have joined have shown so prompt an appreciation of the efforts of their kind friend and benefactor as to hand to the Princess in the purses she has received to-day the sum of £2,200 to form the nucleus of a Junius S. Morgan Benevolent Fund for the benefit of nurses who are less fortunately situated than themselves. I can only hope that the generous example set by Mr. Morgan, whose loss we all deeply deplore, may induce many to come forward and contribute to this excellent benevolent fund which you have spontaneously started to his memory. It will be exclusively devoted to the alleviation of special cases of distress amongst nurses throughout the British Empire, similar to those of which I have given you three typical examples. It is satisfactory to notice further that the Pension Fund is proving of very real assistance to hospitals and kindred institutions which train and employ nurses. Several of these institutions —notably the great London Hospital, Guy's, St. Mary's, and King's College hospitals, as well as others both in London and the country—have become affiliated to the fund, and many pay one-half of the necessary premiums to secure a pension to the nurses in their employ. I understand that the Colonial authorities are taking an interest in the Pension Fund, because all nurses working in the British Empire are eligible to join it, and I trust that very many Colonial hospitals will become affiliated on some such plan as that adopted by the London and Guy's Hospitals and other institutions. No one can do without a nurse sooner or later, and all must hope that the Council will be justified by the result in their belief that these proceedings will cause many hospitals and nurses to join the Pension Fund, and that a great many people will be moved to inquire about it. If this proves to be the case I am certain that the Pension Bonus Fund and the Junius S. Morgan Memorial Benevolent Fund will receive substantial additions from many quarters. It is at any rate satisfactory to be able to record the fact that the success of the National Pension Fund for Nurses has been so phenomenal as to prove that women can and will attend to their own interests, and provide for old age and incapacity when the day for work is over.

The Chairman's Reply.

Mr. Walter H. Burns, Chairman of the council of the Fund, and son-in-law to the late Mr. Junius S. Morgan, said : "Your Royal Highnesses,—It becomes my pleasant duty as Chairman of the National Pension Fund to return thanks to the Princess and yourself for your gracious reception of the nurses this day ; but before doing so I wish to thank you, in the name of the family of Mr. Morgan, for the kind and appreciative words relating to him which are contained in your address. The message of sympathy which you sent to his sorrowing family when he was lying death-stricken at Monte Carlo was a source of deep satisfaction to them, and this additional proof of your good will to the memory of our lamented dead has rendered us, although citizens of a foreign country, most loyal and devoted adherents to your persons as long as our life lasts. I wish also to thank the nurses for the memorial that they have built to his memory. It was so spontaneous, so unexpected, so unsolicited a tribute to one who took the deepest interest in them that it is more solid than if erected with stone and brass, for it is founded upon the kindly feelings of those who had his greatest sympathy. It will endure as long as the National Pension Fund itself, and is a memorial which will be an incentive to his children and grandchildren to continue the work in which he took so much interest. On behalf of his family, and on my own, we hereby pledge ourselves to continue in every way the work which he had so earnestly commenced. Your address, Sir, has been so complete in relation to the present position and progress of the Pension Fund that I feel it would be a waste of time to dwell any longer on that subject, but there is one point upon which I desire to insist in order that my words may reach those whom otherwise I might not attain. While the general public and the nurses have responded most enthusiastically to the scheme for their benefit, we have been a little disappointed in the response from the hospital authorities in seeking affiliation with us. This is due to many reasons; but whether it be because they have funds of their own, or for want of funds, I would call the attention of the governors to the following points: In our system of affiliation the nurses pay one-half of the premium, the hospital the other half; and if by the donations and benefit funds which we have formed we can nearly double the value of the annuity the hospitals will obtain for one-fourth to one-third of its real worth, the advantage that they pay for. I am so sure of the deep interest that the governors take in their working staff that I feel certain it is only necessary to put before them this commercial consideration to lead them to take further steps in affiliating with us. If they do so, the next progress will be to extend our work into the colonies, and form another link in the chain which binds Great Britain to its distant possessions, thus still further developing the idea of which the Imperial Institute, of which you are the wise founder, is the symbol and expression. Now, Sir, I wish to convey, more especially to yourself, the thanks of the nurses for the honour you and the Princess have conferred upon them and the pleasure you have given them this day. The Royal Family of England have always been known for their deep interest in everything which was for the true benefit of their people, but I am sure that there is no movement with which they have been associated more conducive to good than the aid and protection afforded to nurses. The Queen, by the bestowal of her jubilee gifts for district nursing, you and the Princess by consenting to become Patron and President of our Pension Fund, and the Princess Christian, by her interest taken in the British nurses, have all shown that the Royal Family is united in its appreciation of the services rendered by that profession, and in the desire to give it the benefit of their protection. The nurse's life is a peculiar one. Fame and fortune do not follow that calling. All others have their rewards; the nurse has to seek hers in the consciousness of duty well performed and in suffering alleviated. The soldier carries his marshal's bâton in his knapsack; the lawyer, the doctor, the merchant achieve titles and fortune. These we can never give to the nurses, but we can give them thrift and honour. The former our National Pension Fund is designed to develop and encourage; the latter you, Sir, and the gracious Princess have conferred upon them this day. Every nurse who is here will leave these beautiful gardens with her mind filled with gratitude towards yourself, and when they perform their tiresome routine of work in the hospital or in the sick room, they will remember with pride and gratification that their calling has received the hearty endorsement of the first family in the land. I beg to renew my thanks on behalf of the officers of the National Pension Fund and the nurses for your most cordial and generous hospitality this day."

### The Reception.

The following had the honour of being presented to their Royal Highnesses: Mr. and Mrs. Walter H. Burns, Mr. J. P. Morgan and Miss Morgan, Mr. and Mrs. Burdett, Miss Billings, and Colonel Crease, C.B.

The speeches over, the nurses were directed to heavily-laden tables, where substantial refreshments were awaiting them. Her Royal Highness then sank the Princess in the hostess, and came out on the lawn and walked about unattended among the nurses, seeing that none were neglected, and speaking to those who attracted her attention. Her example was followed by her daughters, and the Prince also mixed with the guests. The whole ceremony was characterised not only by princely, but by kindly conduct, and never before had Royalty shown itself in a better light. The band of the Guards was in attendance, chairs were placed under the trees, and no effort had been spared to make the nurses' garden party at Marlborough House a pleasant entertainment. No nurse who was present could possibly have left without a deep feeling of gratitude to the beautiful Princess, to whom the thanks of all were too great for expression by mere applause. A few guests had been invited by their Royal Highnesses "to meet representatives of the first thousand nurses," and these included the Duchess of Beaufort, the Earl and Countess of Rosebery, the Countess of Strafford, Lord and Lady Rothschild, the Earl of Aberdeen, Sir E. Hay Currie, Mr. H. H. Gibbs, Mr. E. A. Hambro, Mr. J. P. Morgan and Miss Morgan, Mr. W. Rathbone, M.P., Mr. and Miss J. Hutchinson, Mr. and Mrs. W. H. Burns, Mr. and Mrs. Burdett, Miss and Miss Olive Burdett, Dr. Bristowe, Dr. Broadbent, Mr. Thomas Bryant, Mr. E. Murray Ind, Mr. P. A. Nairne, Mr. E. Rawlings, Mr. A. C. de Rothschild, Dr. Steele, Dr. Potter, Mr. J. Watney, Mr. Clifford Wigram, Mr. G. P. Pocock, Mr. E. T. Clifford, Sir James and Lady Paget, Sir Andrew and Lady Clark, Dr. Billings, the Misses Billings, the Rev. and Mrs. Dacre Craven, Miss Luckes, Mr. J. R. Lunn, Miss Wilson, Miss Smythe, Mr. Leopold de Rothschild, and Colonel Crease, C.B.

---

### THE CERTIFICATES PRESENTED BY THE PRINCESS.

Specimens of the two forms of certificates presented to the First Thousand Nurses were exhibited in handsome frames, designed by Miss Lillian C. Smythe, which had been tastefully draped with the Danish colours by Mrs. Clifford.

Through the courtesy of the proprietors of the *Graphic* we are able to reproduce the certificates which were presented to the First Thousand Nurses at Marlborough House, on Friday, the 4th instant. It will be seen that there are two kinds of certificates. The first certificate was given to those nurses, the majority, who joined the Fund unaided by any institution; and the second was given the nurses who received some help, on the half premium or some other principle, from the institutions where they worked.

---

### MINOR INCIDENTS.

It is impossible to pass over some of the trifling events of Friday, which, though small, illustrated the spirit of the proceedings. For instance, it was discovered that some nurses had outside the gate a beautiful bouquet for the Princess of Wales, brought all the way from Oswestry. The bouquet was unpacked and brought in, but, as it was quite unexpected, no arrangements had been made for its public presentation, so it was quietly handed to the Princess by Colonel Stanley Clarke after the ceremony was over. This little gift from the nurses was much appreciated by their Royal hostess.

Another small incident was the going astray of one out of the 600 certificates—the wonder was that more were not missing! Well, the nurse who presented herself but received no certificate was of course sadly disappointed. Was she entitled to the certificate? asked the Princess. Yes; the nurse was one of the first thousand. "Then," said the Princess, "wait patiently till your certificate is found." After all the other presentations were made, the lost roll came to light, and the Princess of Wales gave the formerly disappointed nurse a little presentation to herself, which more than made up for previous sorrow. "By little deeds," said an Eastern philosopher, "you can measure a great soul."

Then is it possible not to record the presence of the two dogs—the wire-haired terrier and the favourite pug of the Princess? They have figured in print before, and though they were enticed away to less dangerous positions once or twice, they always returned to the feet of the Princess and ran to and fro between her and her guests. Wise

CERTIFICATE TO UNAIDED NURSES.

A

CERTIFICATE TO INSTITUTION NURSES.

B

deserve to again, on account of the great friendliness they betrayed for nurses. With wagging tails they cheerfully meet, and nearly upset, the advancing squads, and animals! knowing well their welcome was sure with every woman, from the highest in the land to the lowest.

With regard to Thursday night, an amusing story is told of a nurse who, about ten o'clock, announced she had nowhere to sleep, had not asked for lodgings, and had no friends in London. The simplicity of the confession was charming; luckily a hospital matron came to the rescue and proved a hospitable matron, for she carried off this lonely nurse, undertaking to find her some sort of a bed and some sort of a breakfast. As for all the nurses for whom we found lodgings, we cannot but hope they were comfortable, and that no disagreeable incidents marked this pleasant fête.

Perhaps the most astonishing thing which teaches the great lesson of the power of combination was the fact that £2,200 was collected by nurses in a short six weeks, merely through a small paragraph in these pages. The suggestion was made after much consideration. We felt all along that a Benevolent Fund founded by the nurses who had made themselves safe by joining the Pension Fund, would have been very cordially approved by our lamented friend, Mr. Morgan. The nurses took to it at once, letters poured in, the Pension Fund officers set the printing-presses going at full speed, and collecting-books were soon spread all over the country, and even went to the colonies. Think of it, nurses! This is the sort of thing you can do by unity of aim. Would that the whole profession would work together in all things!

### OUR HEARTY THANKS.

And now, dear readers—dear friends many of you have grown to be—we know you join with us in our final chorus of thanks and praise. To the Prince and Princess of Wales for their gracious hospitality and right royal welcome, first and foremost, and with extra heartiness. Also to Colonel Stanley Clarke, C.M.G., and Mr. F. M. Bryant, for kindly helping with the arrangements at Marlborough House. To the Master, Wardens, and Court of Assistants of Merchant Taylors, and to Mr. Faithfull and their staff in general; to Mr. Ernest Shute and Mr. Totton. To the following railway companies for their generous offer of conveying the nurses to and fro for a single fare: The Great Western, Great Eastern, Great Northern, Caledonian, London and North-Western, London, Brighton, and South Coast, London, Chatham, and Dover, Midland, London and South-Western, North Eastern, and North British. Our thanks are also due to Mr. Michelli for the programmes for Thursday night, to Mrs. Michelli for the design which adorned them, and to Herr J. R. Sawerthal for so kindly conducting the band of the Royal Engineers in person. As for the entertainments on Thursday night, Mr. Alfred Izard had charge of the concert given in the drawing-room, whilst Mr. Douglas Beaufort gave musical and ventriloquial sketches in the court room, but we must confess we had not time to enjoy them as we wished: a delightful violin solo, a few verses of a duet, one excellent conjuring trick, and part of a most comical musical sketch—these were all the glimpses we got of the entertainment rooms, so that our readers probably are better critics than we are on these points. Then surely thanks are due to Colonel Crease for coming up from Portsmouth to superintend the drilling, and to Miss Lillian C. Smythe for designing and packing the certificates so prettily. Indeed, the thanks seem endless—the gratitude too great for expression. And surely the reward of all who worked much or little in the cause of the fête was found in the great success which attended the function.

### FRAMES FOR CERTIFICATES.

Specimens of the certificates framed in oak and gilt, with the Prince of Wales' feathers in the centre, were exhibited at Merchant Taylors' Hall and Marlborough House. These frames were designed, with the assistance of Miss Lillian C. Smythe, by Messrs. G. and A. Brown, 24, Newman Street, Oxford Street, W., who have undertaken to supply frames of a similar pattern to nurses at the following specially reduced rates:—For single frames complete with glass, &c., 15s. each. If fifty or more frames are ordered, then they will be supplied to nurses at 12s. each. In the case of nurses residing out of London, Messrs. Brown will undertake to pack and forward them in a case for an additional cost of 2s. 3d. each, making the inclusive cost of the frame, packing, and forwarding, 17s. 3d. in the first instance, and 14s. 3d. in the second.

Nurses attached to the same institution possibly might make better terms as to carriage, &c., if the frames were all forwarded together. Communications should be addressed to Messrs. G. and A. Brown, as above, or to the Manager of the Pension Fund, 8, King Street, Cheapside, E.C.

### Queen Victoria's Jubilee Institute for Nurses.

TO THE EDITOR OF "THE HOSPITAL."

Sir—We have the permission of her Majesty to make public the following statement, and we shall be much obliged if you will kindly insert it in the columns of The Hospital.—We are, Sir, your obedient servants,

ARTHUR L. B. PEILE, President.
WESTMINSTER, JAMES PAGET, RUTHERFORD ALCOCK, Trustees.

St. Katharine's Royal Hospital, Regent's Park, July 2.

Statement as to the past and present position of Queen Victoria's Jubilee Institute for Nurses:

Her Majesty having decided (in accordance with a memorandum dated December 26th, 1887, which was, at her desire, submitted to her by the Duke of Westminster, Sir James Paget, and Sir Rutherford Alcock) to devote the surplus of the Women's Jubilee Offering, amounting to £70,000, for the purpose of forming an institute for the promotion of the nursing of the sick poor in their own homes, appointed a provisional committee to consider the best means of carrying out such a scheme. Her Majesty also determined that the institute should be directly connected with the ancient Royal hospital of St. Katharine, in the Regent's Park, and that its centre for work should be established there.

In their endeavours to give effect to the beneficent intentions of her Majesty, the provisional committee decided that the interest of the fund (about £2,000 a-year) could be most usefully employed in promoting the formation of central institutions, and in assisting institutions already formed, where the training of nurses for the work of the institute could be brought up to the highest possible standard, hoping that the work of tending the sick poor in their own homes by thoroughly-trained nurses would eventually be extended throughout the country.

It was not possible to form at once a new training school in London. The committee, therefore, made arrangements with the Metropolitan and National Nursing Association to train for them a limited number of probationers who should be competent to take charge of nursing the sick poor in their own homes in such districts as may be assigned to them.

A branch centre has been formed in Edinburgh, which is in full working order and is rapidly extending its work in Scotland.

Arrangements are in progress in Dublin by which it is hoped that the work of training nurses for the sick poor will be as successfully carried on.

A Welsh centre has been formed at Cardiff.

Superintendents have been appointed for each of the branch centres, as well as a general inspector, whose duty it is to report to the central body in London respecting the work and efficiency of all nursing associations which are in any way connected with the Queen's Institute.

The work of administration has up to the present time been voluntarily carried on by the members of the provisional committee.

In September, 1889, a Royal Charter of Incorporation was granted, and a council was appointed by her Majesty in February, 1890.

It consists of the following names: Rev. Arthur L. B. Peile (Master of St. Katharine's), president; the Duke of Westminster, K.G., Sir James Paget, Bart., Sir Rutherford Alcock, K.C.B., trustees; the Countess of Rosebery, Lady O'Hagan, the Hon. Lady Ponsonby, Mrs. Theodore Acland, Mrs. Henry Grenfell, Mrs. Craven, the Right Hon. the Earl of Meath, Sir Dyce Duckworth, M.D., Henry Bonham Carter, Esq., William Rathbone, Esq., M.P. (vice-president), John Jaffray, Esq. (Birmingham), Oliver Heywood, Esq., D.L. (Manchester), Rev. Bertram Darley (St. Katharine's).

The council have now taken over the work which the provisional committee set on foot, and are anxiously endeavouring to give permanence to the work which her Majesty has committed to their care. That work is set forth in the charter as being "generally the promotion and provision of improved means for nursing the sick poor." It is manifest that the extension of this national work must depend upon the formation of local institutions, and the maintenance of them, when once started, must be the result of the charitable effort of those who live in the neighbourhood.

The council of the Queen's Institute will readily co-operate with those who desire to form such nursing associations, whether in the country or in towns, but the interest of the fund at their disposal will only suffice for such purposes as fall within their province as a governing body.

By the generous gift of £5,000 from Mr. Tate and £1,000 from Mr. Rathbone, the council are enabled to extend their immediate efforts to assist new nursing associations already in existence, provided that they are able to adopt the conditions which they have laid down. Speaking generally, those conditions are:

1. That nurses shall have had at least one year's training in an approved general hospital or infirmary.

2. That they should have approved training in district nursing for not less than six months, including maternity nursing.

3. That nurses in country districts must have at least three months' training in midwifery.

The council are of opinion that the advantages of affiliation will be:

1. To bring associations into connection with the institute which bears her Majesty's name.

2. To assist in raising the standard of thoroughly trained nurses for the poor.

3. To entitle affiliated associations to such aid as the council may be able to give.

4. To entitle nurses attached to affiliated institutions who satisfactorily fulfil the conditions of thorough training, efficient work, and unexceptionable conduct to have their names submitted to her Majesty to be placed on the roll of Queen's nurses.

The council do not desire to interfere with the management of any existing association, but only to satisfy themselves that the work of training nurses which is being carried on is of such a character and standard as will admit of its being affiliated to the Queen's Institute if it be so desired. A work of this magnitude can only become fully established gradually, but the measure of success which has been attained up to this time encourages the council to hope that so valuable an institution will be acknowledged by the public as one which promises to confer lasting benefits in many ways upon the poor in the land, and which should be made worthy of the circumstances connected with its foundation.

### Sketches of Insanity.

By Francis H. Walmsley, M.D. (Senior Assistant Medical Officer of Leavesden Asylum).

### II.—CAUSATION OF INSANITY.

" Reason's whole pleasure, all the joys of sense,
Lie in three words, health, peace, and competence.
But health consists with temperance alone;
And peace, oh, virtue! peace is all thy own."—Pope.

Insanity is one of nature's penalties for her broken laws. It is not caused by the chance shafts of some mysterious agency.

It may be affirmed that no cause whatever, no matter how disturbing, will upset the sound mind allied with the sound body. Great mental work can be well borne if there be a due observance of the laws of health.

The most potent causes of insanity are hereditary transmission and alcoholic intemperance.

*Hereditary Transmission.*—Were it possible to trace the family history it would probably be found that no less than one half of all occurring cases of insanity are due to inherited taint. Although the parents may not have been insane, insanity may have existed in the grandparents and reappeared in the grandchildren, skipping a generation.

As examples of hereditary taint, there may be cited the case of a noble family of Hamburg remarkable from the time of the great grandfather for their military talents, in which all the male descendants were attacked with insanity at the age of forty. Only one remained, an officer; he was forbidden to marry, the critical age arrived, when he too went mad. Again, the case of a family in which the father, two brothers, two sisters, two cousins, and an aunt, all were insane.

As instances of hereditary suicidal insanity may be quoted the case in which the grandmother, mother, and grandchildren were the subjects of suicidal melancholia; and another in which the father, who was of a taciturn disposition, had six children, five sons and a daughter, all of whom at different times, and by different means, committed suicide, impelled thereunto by trifling causes.

*Alcoholic Intemperance.*—One-fourth of all occurring cases of insanity are due to drink. Intemperance of parents generates mental morbidness in their offspring, which may manifest itself in the form of hysteria, epilepsy, idiotcy, or insanity.

*Other Causes are*:—Epilepsy, excesses of all descriptions, the developmental and critical periods of life, the puerperal state, bodily diseases—as apoplexy, syphilis, phthisis, etc.—anæmia, with defective nutrition of brain; domestic trouble, loss of relatives or friends, adverse circumstances, worry, religious excitement, love affairs, fright, shock, injudicious marriages, poverty, with all its attendant physical evils of insufficient food, the care for to-morrow, and insanitary conditions generally—in a word, too much of the gloom and too little of the light and sweetness of life. For the most part those who are affected by such causes are, by reason of hereditary taint, already predisposed to insanity.

*Sex.*—Probably more males than females become insane, though the difference in the numbers is very slight.

*Age.*—No age is exempt from liability to insanity. This liability increases with the progressive development of brain and mind—the period of greatest mental and bodily activity—consequently insanity is most frequently met with between the ages of 25 and 45 or 50.

*Education.*—The importance of a judicious training of the young, especially during early development, cannot be over-estimated. Due regard should be paid to the mental capacity of each child.

In connection with the question of the higher education of women and their introduction to the deeper studies, Herbert Spencer says:—

"The mental powers so highly developed in woman are in some measure abnormal, and involve a physiological cost which her feminine organizations will not bear without injury more or less profound."

The educational system should be constructed with the view of developing and strengthening the whole organization, both mental and physical.

*Marriage.*—Very early marriages should be discountenanced. The health of the mother, and consequently of the offspring, has a less chance of being deteriorated by delaying the woman's age of marriage to 25 or 26 years.

*Consanguineous Marriages.*—The frequent intermarriage of close relations tends sooner or later to insanity. First cousins should not marry unless both they and their ancestors have been free from insanity. It is better to prohibit marriages between blood relations down to, or even including, second cousins. The celibacy of the insane must be enforced. Those in whom the insane temperament is well marked should not marry; such unions are fraught with danger to the individuals, to the offspring, and to society.

*Uncivilised or Barbarous Peoples.*—It is stated that the lower nations are in the scale of education and civilisation the less the frequency of insanity among them. The Inspector-General of the Insane in New South Wales says that mental disease would appear to have been a very rare affection among the aborigines of Australia while they were in their primitive and uncivilised condition, and the manner in which they dealt with the few cases which did arise was of the most drastic nature. "If the lunatic was violent or aggressive he was promptly slaughtered; if melancholy, he was allowed, if so disposed, to commit suicide; if demented and helpless, he was allowed to die, and only when quiet and peaceable and when his erroneous ideas did not result in offensive acts was he allowed to continue in the tribe." On the other hand, Emin Pacha records that the wild tribes of Central Africa are not free from what are often thought to be the diseases of modern life. Epilepsy and insanity are common among them. Drunkenness is not, as frequently stated, introduced by Europeans, but is one of the favourite vices of the primitive negro.

*Proportion of Recoveries.*—Of eleven persons attacked by insanity, six recover, five die sooner or later during the attack; of the six who recover, not more than two remain well during the rest of their lives, the other four sustain subsequent attacks, during which at least three of them die.

*Is Insanity Increasing?*—Insanity has a tendency to die out like other diseases. The numbers of our lunatics would be tenfold what they are but that we have extinction—or a return to a healthy type. This tendency to revert to the normal is invariably manifested in the offspring, especially under improved conditions.

From available statistics it cannot be inferred that there is any actual increase of occurring insanity. The increase in the number of insane persons is more the result of accumulation than an actual increase in the number of new cases in proportion to the population, and it is cheering to note that in the cases recently admitted the form of insanity does not show any sign of becoming of a worse type than formerly.

We may indulge in the hope that as the healing of social calamities progresses, the call for the erection of the "palaces of desolation," so pathetically alluded to by Lord Rosebery when recently laying the coping stone of another metropolitan asylum, will become less and less imperative.

*(To be continued.)*

### SILENCE.

"You are bound by no Hypocratic oath, yet your calling is one which necessitates the observance of a silence as sacred as that which binds the physician and the priest. Let a seal as inviolate as that of the confessional be upon your lips. Whatever you may hear in one family, let it be upon your honour as to one bound by a vow. So surely as one among you becomes known as a bearer of gossiping tales, so surely that one's day of usefulness draws to an early and unregretted close."—*Dr Charles Simmons.*

### A Day Off.

I am sitting on the garden roller, in a sunny nook formed by the projection of a large bay-window, and before me spreads a prospect of miles of wooded hillsides. On the bank in front the pheasant-eyed narcissi shake their sweet heads, and the amber sprays of the berberis bow gracefully in the western wind. A blackbird hops inquiringly near me, and re-assured on the subject of cats proceeds to seek a worm for luncheon. On the warm breeze comes the smell of lately cut grass, and the humming of the insects in the ceanothus bushes. How restful it is, this smiling sweet-scented morning.

And only yesterday when I returned from my case with aching back and tired brain, I said to myself that nothing should take me from home for at least twenty-four hours. But I found waiting in my London eyrie a note which ran: "Come, little nurse, and spend the Sunday with us; the forest has put on its golden-green frock, and the nightingale has commenced to sing. Come, and we will not try to amuse you." It was the last sentence that decided me. I love some of my friends dearly, but I only stay with them when I have had a month at the sea and a course of tonics, then I can stand with resignation the calls on people I don't want to know, and the drives to places I don't want to see. I can hear with serenity the performances of the village band, and appear meek and grateful, as becomes my position, when patronised by the vicar, and instructed in nursing by ladies who have attended ambulance lectures; but when I go to the country for rest I want just to breathe the air and gaze at the cabbages. I delight in cabbages, they are so fresh, so vigorous, they spread out their veined leaves to the sun, and their great succulent hearts are bursting with fulness of life. If I were a grand physician I should say to my nervous patients, "Go out among the cabbages, meditate upon them and grow strong, but mind you change your boots when you come in."

Once upon a time there lived a Princess; of course she was young and lovely, and, what is better, she was strong and healthy, with a magnificent set of nerves; she never had neuralgia, or anæmia, or St. Vitus' dance, or any other uncomfortable ailment; she was so bright, so bonny, so full of love for everybody, that all the world sang her praises. Now a little way off lived another Princess who had a sluggish liver and a defective digestive apparatus, therefore she was ugly and ill-tempered, and hated with a deadly hatred her charming and wholesome neighbour. So she took some brimstone and arsenic, or whatever was the fashionable wizard's mixture of the period, and stirred it over the slow fire in the largest copper preserving-pan she could borrow, then three times she drew a magic circle with the aid of a pot lid, and three times she muttered the awful word Daumschrauben, and immediately the beautiful Princess shrivelled up and died.

And out of her grave sprang the cabbage, which gives its great heart to man and to beast.

As for the wicked Princess, the fumes of the magic brew greatly improved her complexion, and not having the perfections of a model neighbour to aggravate her, she regained her peace of mind and lived happy ever after.

I do hope Mr. Editor won't be offended because there is no moral to this tale, but I read once in a high-class review that the noblest and best work in art and literature is that which follows nature most closely, so I have followed nature and have not upset my ugly Princess into the preserving-pan as I had intended. The bells begin to ring from the little tower close by and rouse me from my dreaming on the old stone roller; the sight of a basket by my side reminds me that I have undertaken to feed the chickens, and I walk slowly through rows of my pet vegetables to the little meadow at the end of the garden. I am not much interested in the fowl, which are of a pure and hideous breed, and wear a half-mourning suit of speckled black and white, so I feed them merely as a matter of duty and watch through the trees the people strolling up the path to the little church over the way.

The architecture of this church is bewildering. It appears from the position of the tower that the ringers perform their duties in the central aisle. The building is quite modern; the architect has created a new style, and produced a substantial pigeon-house, with wings on three sides and an outhouse on the fourth. Query—Is this the style of architecture that we are told will evolve from the social needs of the people? The bells have stopped, and I can hear the note of the cuckoo in the distance; a white butterfly settles on the wild parsley growing near: it is the first I have seen this year, so I shall eat white bread till spring comes round again, which does not seem to be such an advantage as in olden days. Of course, everybody knows that a brown butterfly means brown bread.

In the evening I stroll up a hill into the forest and watch the sun as he sinks in a crimson glory behind High Beech. As he goes slowly down, the red cloud thins and softens, spreading itself in patches of pale rose colour over a background of delicate greenish-blue, which darkens into grey. Somewhere among the trees the nightingale is trying her voice, and some horses that have been turned loose in the forest come within a short distance and ask, as plainly as eyes can express, whether I am going to give them bread or a stone; they are probably accustomed to both, as the moods of holiday-makers are uncertain. I try to make friends with them, and have nearly succeeded, when a noise of breaking twigs startles them and they move off. The sounds are made by a youth, evidently an East-ender, who comes out of the thicket whittling a stick. A girl follows him, her pale sharp face half lost in a hat of true Whitechapel style. With one hand she holds up her draggled skirt and a shabby bag, the other supports a puny baby of about five weeks old. As they go up the hill, the woman some feet behind, the weak head of the infant nods violently with every step of the weary mother, and I wonder it does not cry. One thing I determine as I go homewards, and that is, if ever I marry a Whitechapel man and he leaves me to toil behind with the bag and the baby I shall always carry a grappling hook to attach myself to him when going up hills.

### THE NURSE!

*(Not the Angel.)*

Who is it comes, a perfect pest,
At five a.m. and breaks my rest,
Disturbing me in my warm nest?
The Nurse!

Who draws my locker to my bed
And puts thereon tea, egg, and bread,
And says "Wake up, you sleepy head"?
The Nurse!

Who to remove superfluous dirt,
A basin brings with orders curt,
"Sit up and wash, take off your shirt"?
The Nurse!

Who seats me on a cold, cold chair,
And makes my bed while I wait there,
Feeling as cross as any bear?
The Nurse!

Who rushes round till half-past eight,
Dusting at a terrific rate,
And scolding if the least bit late?
The Nurse?

Who goes when things are all put right,
And leaves me grinning with delight,
But dreading still the coming night?
The Nurse!

King's College Hospital.

### Beef-Tea.

Dr. Arthur Luff, lecturer on Medicine at St. Mary's and assistant physician to the North-West London Hospital, contributes to the *Lancet* a paper on the composition of beef-tea, and its value as compared with some other preparations of beef. Dr. Luff has made analyses of several preparations. 1. Strong beef-tea, made by finely chopping one pound of rump steak, mixing it with one pint of water, and infusing for six hours in an earthenware vessel kept in boiling water, the beef-tea being not strained, but on standing poured off from the portion of meat debris that had settled, some particles of meat debris still remaining in suspension. 2. Beef-tea prepared by adding one dessert spoonful of concentrated Bouillon Fleet to a large cupful of water. 3. Beef-tea prepared by dissolving one teaspoonful of Kemmerich's peptone of beef in a large cupful of water. 4. Beef-tea made by dissolving a quarter of a teaspoonful of Kemmerich's extract of beef in a large cupful of water. The analyses show that only a small quantity of proteid or albuminous matter is present in any of the beef-teas examined. Also that the beef-teas made from Bouillon Fleet and from Kemmerich's peptone of beef are throughout stronger in composition than the best beef-tea made from rump steak. The beef-tea made from one quarter of a teaspoonful of the Kemmerich extract is slightly weaker than ordinary beef-tea. Dr. Luff is therefore of opinion that we have in beef-tea, prepared either from Bouillon or peptone of beef, a preparation which is both stronger as regards important constituents, and cheaper than ordinary made beef-tea. He remarks, "the comparative cheapness is easily understood when we consider the great difference of price of beef in this country and in America." Dr. Luff says that the stimulant tonic and dietetic properties of beef-tea depend mainly on the peptones, which are taken up by the intestinal mucous membrane and transformed by it into serum albumen. Recently it has been denied that beef-tea possesses much nutritive value, but even if this is the case Dr. Luff states its use as a stimulant and tonic cannot be denied. "On our exaggerated estimate of the value of beef-tea" is the title of a paper published some time ago (*British Medical Journal*, January 26th) by Mr. Laffan. This authority observes that we may accept the statement of Liebig that to extractives and salts is due all the value beef-tea possesses, and that it neither economises carbon for our temperature nor nitrogen for the substance of our tissues. Mr. Laffan also quotes Mons. Sée, who tells us that beef-tea contains very little albumen and very little carbo-hydrates. Mr. Laffan thinks that milk and minced raw meat are better resources than beef-tea. Dr. Tomkins, of Clifton, also wrote an article on this subject. He thinks beef-tea as a food ranks low, and he advises raw beef-tea and peptonised milk. The use of beef-tea is, however, so general, and the public have so much confidence in it, that it will be long before it is abandoned, unless something more in its disfavour can be advanced, and the benefit derived from it by the sick becomes less apparent than it now is. The public do not care how it acts so long as when sick they gain strength from it.

In connection with the above may be mentioned an easy method of giving beef-tea or other aliment *per rectum*, contrived by Mr. Jones-Humphreys, of Cemmaes. The apparatus consists of a small funnel, a glass tube about four inches long, which is attached to the funnel by a piece of elastic tubing, and an ordinary flexible catheter, which is attached to the lower end of the glass tube. The atmospheric pressure is sufficient to force any fluid slowly into the rectum, and Mr. Jones-Humphreys has never noticed any return; while the rate the fluid passes into the rectum can be watched through the glass tube. The plan is exceeding simple, the patient being able to pass the catheter into the bowel without pain. The apparatus is cheap, easily made, and readily cleaned. The most inexperienced person may use it. And the fluid being slow in its passage it is more likely to be absorbed. Doubtless this is an improvement on the old enema syringe, the use of which often results in the excitation of the bowel, and the return of the material injected, also an improvement on the funnel and rectal tube, which being of greater calibre than a catheter, is liable also to irritate the bowel.

### Everybody's Opinion.

[*Correspondence on all subjects is invited, but we cannot in any way be responsible for the opinions expressed by our correspondents. No communications can be entertained if the name and address of the correspondent is not given, or unless one side of the paper only be written on.*]

### WORDS OF THANKS.

Nurse Wilson, Royal Infirmary, Preston, writes: As "One of the first thousand" I wish to render my most sincere thanks to our gracious Prince and Princess for the honour bestowed upon us; it has been honour upon honour, and the visit to Marlborough House is an event which can never be forgotten by us. I would also thank most heartily all those gentlemen who have worked so energetically on our behalf; they must have devoted the whole of their precious time to the preparations required for such an honourable event. We cannot possibly thank them all sufficiently for their extreme kindness. Not one thing seemed forgotten which could add to our comfort and enjoyment, and for we provincial nurses it was a great boon having our railway fares reduced, thanks to the railway companies for their generosity in also extending our pass for a week.

### A HOME OF REST.

A. E. S. writes:—Miss Louisa Twining writes as usual with sound common sense; perhaps I think so because I so cordially agree with her. Nurses are grateful for sympathy in their work, always hard (often dirty and disgusting), but they do not want *charity*. With Miss Twining I hope (if collected) £15,000 will be spent to more advantage than in building a "Home of Rest." When our holiday comes we want to be out of the sight and sound of *nurses*, as well as patients. We want, at least once a year to remember what "home life is like." As I before wrote, few nurses are without a friend who can take them in for a fortnight. I have talked this subject over with many nurses and though all agree in gratitude for what is really a kind thought, they had much rather (if homeless and friendless) take a lodging where they please. My nurses have always three weeks, if possible, a month. Shorter hours and better wages to enable nurses to save is more to the point than a "Home of Rest."

### Appointments.

[It is requested that successful candidates will send a copy of their applications and testimonials, with date of election, to The Editor, The Lodge, Porchester Square, W.]

St. Bartholomew's Hospital, Chatham.—Miss Mary Cobbold has been appointed matron of this hospital, and will be able to choose between the military society of Chatham or the clerical society of Rochester. Miss Cobbold, trained at Middlesex as a Lady Probationer, and has lately acted as matron of the Caterham Cottage Hospital. Her testimonials are excellent, and we hope to watch with pleasure her future progress. Miss Cobbold is a sister of Dr. Cobbold of Chiswick.

Essex and Colchester Hospital.—Miss Mary E. Brough was on April 2nd appointed Matron to the Essex and Colchester Hospital. She joined the St. Albans Diocesan Nursing Institution, Witham, in 1882, and was trained by it at the London Temperance Hospital, Hampstead Road, afterwards working for the same institution as private nurse until March, 1888. The last two years she was on the staff at the Radcliffe Infirmary, Oxford, as charge and private nurse.

### Notes and Queries.

### Queries.

(25) *Egyptian Decorations.*—Would someone kindly send a list of the nurses decorated for service during the late Egyptian war, and say what the decorations were?—*Old Naval Nurse.*

### Answers.

*B.T.*—There is no charge for insertion.

"*Doris.*"—Get "The Management of Children" by A Mother. Published by Churchill.

*National Pension Fund.*—Nurse Pantau is eligible, and should write to the Secretary of the Fund, 8, King Street, Cheapside, E.C.

*A.C.S.*—Apply to Winchester County Hospital or the Chichester Infirmary. London hospitals do not take probationers under 25 years of age.

*Miss E.*—Perfect spelling is not necessary to becoming a nurse; there are no mistakes in your letter. Wait till you are 21 and then apply at Bristol or Glasgow Hospital for Children.

### Vacancies.

The following vacancies are announced:

### Matrons.

Dinorwic Hospital, Llanberis.

Kent County Asylum; £85.

Tunbridge Wells Hospital; £50.

### Superintendents.

Assistant Matron, Paddington Infirmary; £30.
Assistant Secretary, New Hospital for Women.
Night Nurse, Greenwich Union; £30.

### Nurses.

Alexandra Hospital for Children, Brighton; £25.

Barton Regis Union; £20.

Birmingham Eye Hospital; £25.

Bishops Stortford Nursing Institution; £25.

Blackburn Infirmary; £27.

Blackheath Institution.

Borough Fever Hospital, Brighton; £24.

Brixton Institute.

Carlisle Infirmary; £24.

Children's Hospital, Cardington, Beds.

Croydon Hospital; £25.

Darenth Asylum; £16.

Dumfries Infirmary; £21.

Frome Nurses' Home.

Glamorgan Infirmary; £24.

Greenwich Union; £37.

Hanover Institute for Nurses; £25.

Highbury Institute.

Levick Institution.

Maghull Home; £20.

Metropolitan Convalescent Home (temp.); £18.

Middlesbrough Fever Hospital; £30.

Margate Sanatorium; £24.

Newcastle Infectious Hospital; £30.

New Winchester Union; £20.

Northern Fever Hospital; £22.

North-Western Fever Hospital; £30.

Nurses' Institute, Jersey; £25.

Poplar and Stepney Sick Asylum; £17 10s.

Royal Chest Hospital; £22.

Scarborough Institute.

Sheffield Nurses' Home.

Shoreditch Infirmary; £22.

St. Mark's Hospital for Fistula; £30.

St. Cuthbert's Poorhouse Hospital, Edinburgh; £25.

St. Pancras Workhouse; £20.

St. Peter's Hospital for Stone, &c.; £24.

Stockton-on-Tees Home.

Swansea and South Wales Nursing Institute; £25.

Western Fever Hospital; £30.

Windsor Royal Infirmary; £20.

Workhouse Infirmary Nursing Association.

### District Nurses.

Birmingham Nursing Society.

Bristol Nurses' Society.

Hulme District Nurses' Home.

Manchester Sick Poor Institution; £25.

Pembury; £50.

### Probationers.

Albert Edward Infirmary, Wigan.
Bedford General Infirmary.
Central Ophthalmic Hospital.
Cottage Hospital, Hammerwich.
Middlesbrough Fever Hospital.
New Hospital for Women.
Stamford Infirmary.
West Kirby Children's Home.

### Amusements and Relaxation.

### SECOND QUARTERLY WORD COMPETITION.

Commenced July 5th, 1890, ends Sept. 27th, 1890.

Three prizes of 15s., 10s., 5s., will be given for the largest number of words derived from the words set for dissection.

Proper names, abbreviations, foreign words, words of less than four letters, and repetitions are barred; plurals, and past and present participles of verbs, are allowed. Nuttall's *Standard* dictionary only to be used.

N.B.—Word dissections must be sent in WEEKLY to 140, Strand, W.C., arranged alphabetically, with correct total affixed.

The word for dissection for this, the SECOND week of the quarter, being

"HENLEY."

| Names. | July 3rd. | Totals. |
|---|---|---|
| Adeline | — | 149 |
| Patience | 94 | 716 |
| Lightowlers | — | 335 |
| Canary | — | 86 |
| M. E. S. | — | 37 |
| Ambition | — | 36 |
| M. W. | — | 217 |
| Esperance | — | 218 |
| Judy | — | 37 |
| Reldas | 97 | 705 |
| Merenda | — | 29 |
| Lucy Locket | — | 40 |
| Camellia | — | 30 |
| Qu'appelle | 91 | 662 |
| Tinie | 95 | 722 |
| Nurse Mildred | — | 190 |
| Coralie | 89 | 690 |
| Gladys | — | 409 |

| Names. | July 3rd. | Totals. |
|---|---|---|
| Agamemnon | 99 | 717 |
| G. E. J. C. | — | 34 |
| S. E. A. | — | 24 |
| Jenny Wren | 86 | 645 |
| Multum in parvo | — | 32 |
| Ecila | — | 39 |
| Weta | — | 259 |
| Henri | — | 161 |
| Holland | 91 | 702 |
| T. J. | — | 111 |
| Nurse Isabel | — | 17 |
| Nurse Faith | — | 9 |
| E. S. Z. | — | 10 |
| G. P. | — | 10 |
| Stumps | — | 33 |
| Bolton | — | 8 |
| Jesmur | — | 58 |
| Ran | — | 17 |

### Results of First Quarterly Word Competition.

*First Prize*, 15s., is awarded to Tinie (Miss Todd, Newbury).

*Second Prize*, 10s., is awarded to Agamemnon (Mr. H. H. Hull, Queen's Road, Weybridge).

*Third Prize*, 5s., is awarded to Patience (Miss Calisé, Craven Road, Newbury).

### Notice to Correspondents.

N.B.—Each paper must be signed by the author with his or her real name and address. A *nom de plume* may be added if the writer does not desire to be referred to by us by his real name. In the case of all prize-winners, however, the real name and address will be published.

Competitors can enter for *all* quarterly competitions, but no competitor may take more than *one first prize* during the year.

## Figures and Tables

**Figure f1:**
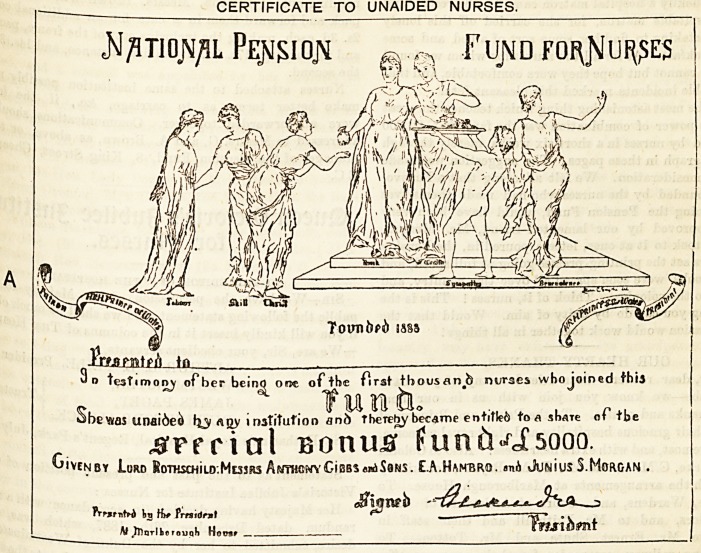


**Figure f2:**